# Values mediated emotional adjustment by emotion regulation: A longitudinal study among adolescents in China

**DOI:** 10.3389/fpsyg.2023.1093072

**Published:** 2023-03-28

**Authors:** Ping Liu, Bibo Mo, Panpan Yang, Dan Li, Shihong Liu, Dan Cai

**Affiliations:** ^1^Department of Psychology, Shaoxing University, Shaoxing, Zhejiang, China; ^2^Department of Psychology, Shanghai Normal University, Shanghai, China

**Keywords:** self-transcendence values, loneliness, depression, emotion regulation, adolescents

## Abstract

Values have a direct impact on adolescents’ mental health. However, its potential mediated mechanism has received little attention. A 1-year longitudinal survey design was used to explore the mediating role of emotion regulation in the relationships between self-transcendence (vs. self-enhancement) values and emotional adjustment among adolescents. Participants were 863 senior school students from Shanghai and Qingdao, Shandong Province. Data on self-transcendence and self-enhancement values, loneliness, depression, and emotion regulation were collected at 2019 and 2020 by using self-report measures. The results showed that (1) adolescents’ endorsement with self-transcendence values decreased and self-enhancement values increased; compared to adolescents in Qingdao, adolescents in Shanghai were more depressed, (2) emotion regulation only mediated the effect of self-transcendence values on loneliness, and (3) not only the relationship between self-transcendence values and depression, but also the relationships between self-enhancement values and loneliness and depression were suppressed by emotion regulation. The study may provide more empirical evidences for the benefits of self-transcendence values and may also give more references on how to improve adolescents’ emotional adjustment.

## Introduction

Values are abstract and desirable goals which reflecting what is important to us (Schwartz, 1992). Adolescence is an important period to form and develop one’s values (Inglehart, 1978), previous studies found that values which adolescents endorse have a significant affect on their psychological adjustment and well-being (Bojanowska and Piotrowski, 2018; Liu et al., 2021). However, how values and emotion adjustment are linked is unclear. According to the General Aggression Model (Anderson and Bushman, 2002) and the Self-centeredness/Selflessness Happiness Model (Dambrun and Ricard, 2011), the current study seeks to address this gap by examing the mediating role of emotion regulation in the relationship between values and emotional adjustment, which may be helpful for intervention for values and mental health among adolescents.

### Theory of human basic values and emotional adjustment

Over the decades, there were many different constructs and theories about values, such as the equality freedom model of ideology proposed by Rokeach (1973). Up to date, the most comprehensive and commonly used one is the model of Human Basic Values proposed by Schwartz (1992). The model assumptions have been extensively studied within different samples and in over 70 countries (Schwartz and Rubel, 2005). In Schwartz’s value theory, there are 10 values which can divided into four higher-order dimensions. According to the motivation the ten values expressed, the values system is organized by a circular continuum. On the adjacent sides of the circle, the values express compatible motivations and usually drive individual to show similar behaviors. However, on the opposite sides of the circle, values express conflicting motivations and usually make individual show opposite behaviors (Cieciuch et al., 2015). As one of the pairs of values with conflicting motives, self-transcendence values include benevolence and universalism values, which express concern for well-being of others and the whole world. In contrast, self-enhancement values include power and achievement values, which express concern for self-interest.

Schwartz (2014) noted that researchers can determine the number of values and the classification of value sets based on their research interests, the type and instrument of analysis used, and the population sampled, as long as the order of the values in circle model is kept constant. The present study focuses on self-transcendence and self-enhancement values. First, the two values are more closely related to emotional adjustment among adolescents. Specifically, self-transcendence values are related to higher-level happiness (Bojanowska and Piotrowski, 2018), less loneliness and depression (Liu et al., 2021), which are important indexes for measuring adolescents’ emotional adjustment (Al-Yagon, 2011). More important, with the rapid development of society and economy, adolescents have become more independent and competitive and pay more attention to personal interests (Chen et al., 2012), which is often accompanied by negative emotions such as anxiety and frustration (Twenge, 2015). Therefore, the study of self-transcendence (vs. self-enhancement) values is also in line with the current development of adolescents, and may help to meet the urgent need to alleviate adolescents’ negative emotions and promote their emotional adjustment.

### The relationships between values and emotional adjustment: The mediating role of emotion regulation

As the above stated, although self-transcendence and self-enhancement values are closely related to emotional adjustment among adolescents, less studies have explored how self-transcendence values prompt adolescents’ healthy growth. Emotion regulation, which refers to the attempts individuals make to maintain, inhibit and enhance emotional experience and expression (Rottenberg and Gross, 2007), may be an important mediating variable. Effective emotion regulation not only promotes one’s mental health and well-being (Haines et al., 2016; Preece et al., 2021), but also motivates one to be more altruistic in social interactions (Song et al., 2018). More important, adolescence has always been called “stormy period,” and it is one of the main characteristics of adolescents with large emotional fluctuations (Casey et al., 2010). Thus, mature emotion regulation is also very important in adolescence.

To our knowledge, previous studies have less examined the relationship between values and emotion regulation. However, some theories as follows may offer a better explanation. The General Aggression Model (GAM; Anderson and Bushman, 2002) states that values as an important individual factor guide aggressive behaviors. Previous studies demonstrated that self-transcendence values are negatively related to aggression, but self-enhancement values are positively associated with aggression among adolescents (Benish-Weisman, 2019; Benish-Weisman et al., 2019). In addition, individual factors further influence aggressive behavior through internal states such as emotions. If negative emotions such as anger and jealousy are not effectively regulated, adolescents may be more aggressive (Strayer and Roberts, 2010). Therefore, values may predict aggression through emotion regulation, and there may be a strong association between values and emotion regulation.

Similarly, the Self-centeredness/Selflessness Happiness Model (SSHM; Dambrun and Ricard, 2011) holds that self-centeredness and selflessness are two qualitatively distinct dimensions and relate to two types of happiness, respectively. Specifically, if one focuses on the self-enhancement values, he or she may experience more fluctuating happiness, which is characterized by the alternation of transitory pleasure and afflictive effects (e.g., hostility and frustration). If one endorses the self-transcendence values, by contrast, he or she may experience more authentic-durable happiness, which is characterized by a state of durable peace and feeling of harmony. Such harmonious connection relies less on positive or negative feedback from the environment, and more on the one’s internal resources, such as mental resilience and emotional regulation, to cope with all experiences of pleasure and pain. In addition, self-transcendence values are associated with durable happiness through emotion stability (Dambrun, 2017), and Liu et al. (2022) also stated that it is necessary to examine the effect of self-transcendence values on emotion regulation to provide new perspectives for understanding the relationship between self-transcendence values and durable happiness. Thus, it is speculated that values may be closely linked to emotion regulation.

As mentioned above, individuals who endorse the self-transcendence values care for the interests and well-being of others and try to connect with others and society in harmony (Dambrun and Ricard, 2011). To maintain friendly social relationships, they may be more proactive in regulating their emotions and preventing inappropriate emotional expressions from negatively affecting interpersonal relationships. In contrast, individuals who endorse the self-enhancement values are more concerned with personal interests. When encountered information that is potentially threatening to their positive self-image, they may often react defensively, such as failing to regulate and control their emotions and expressing their hostility and anger unabashedly (Tamir et al., 2016), regardless of whether these emotions cause harm to others. In summary, we hypothesize that person who endorses the self-transcendence (vs. self-enhancement) values may have proper emotion regulation ability, and then may have better emotion adjustment (i.e., less negative emotions).

### The current study

**Figure 1:**
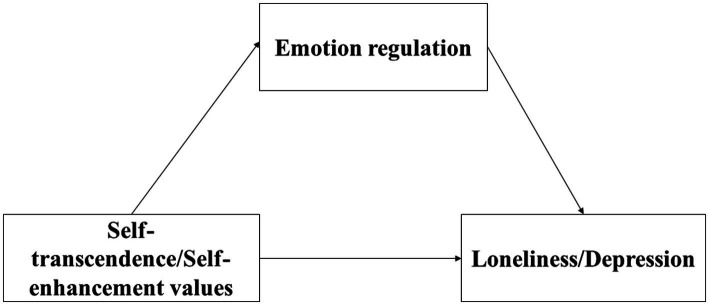
The mediation hypothesis model.

Although previous studies have suggested that values have an important influence on emotional adjustment, cross-sectional design cannot reveal the causal relationship between variables; and less study has investigated its potential mediation mechanism. Moreover, some theories offer a better explanation to understand values and emotion regulation, however, the relation between them needs to be further examined. Thus, a 1-year longitudinal design is used to explore the mediating role of emotion regulation between the two values (i.e., self-transcendence and self-enhancement) and emotional adjustment (i.e., loneliness and depression) in Chinese adolescents. The mediation hypothesis model is shown in Figure 1.

## Methods

### Participants

Participants in senior schools from Shanghai and Qingdao, Shandong Province were selected. Shanghai, locates on China’s central eastern coast at the mouth of the Yangtze River, is an international metropolis. Qingdao is located on the eastern coastal areas of Shandong Province, which is the origin of Confucian culture. There were 939 students in Grade 1 (371 in Shanghai and 568 in Shandong) at Time 1. From the original sample, there were 863 students at Time 2, 330 in Shanghai (*M*age = 17.15 years, SD =. 75), and 533 in Qingdao (*M*age = 17.35 years, SD =. 80). There were 10 and 24 classes in Shanghai and Shandong samples, respectively. Ten classes in Shanghai were surveyed and 11 classes in Shandong were randomly selected, with approximately 45 students in each class. The *χ*^2^-test and *t*-test results showed that there was no significant difference between adolescents who participated at T2 and those who did not on gender (*χ*^2^(1) = 2.07, *p* = 0.15), age (*t* (937) = −0.07, *p* = 0.947), T1 self- transcendence (*t*(894) =. 20, *p* = 0.844), T1 self-enhancement (*t*(894) = 1.69, *p* = 0.091), but there was significant difference between the two groups on T1 emotional regulation (*t*(925) = −4.02, *p* < 0.001). We gave a gift to participants as a reward for their participation after the survey.

### Measures

*Values*. We used the Portrait Values Questionnaire (PVQ; Cieciuch and Schwartz, 2012) to assess participants’ values. The PVQ included 40 items, and by using an implicit way, each item described a short verbal portrait, which reflecting a person’s goals or aspirations that point to the importance of a value type. We only used the self-transcendence (including 10 items) and self-enhancement values (including 7 items) subscale. By using a 6-point (1 = not like me at all, 6 = very much like me) scale, participants should rate how similar was the described person to them (e.g., “*It is important for him/her to help the person around him/her. He/she wants to care about them and make their lives happy*” describes a person who endorses with the self-transcendence values. “*Success is important to him/her. He/she likes to make a good impression on others*” describes a person who endorses with the self-enhancement values.). To control for response tendency, we used a previous adjustment method (Schwartz and Bardi, 2001; Gu and Tse, 2018) to calculate the scale scores. Previous study in Chinese adolescents had proved the PVQ was reliable and valid (Liu et al., 2021). For the self-transcendence values, the internal reliabilities were .75 and .80 at Time 1 and Time 2 respectively; in addition, T1 McDonald’s Omega (Ω) = 0.753, T2 McDonald’s Omega (Ω) = 0.799; T1 CR (Composite Reliability) = 0.82, T1 AVE (Average Variance Extracted) = 0.32; T2 CR = 0.85, T2 AVE (Average Variance Extracted) = 0.36. For the self-enhancement values, the internal reliabilities were .72 and .73 at T1 and T2 respectively; in addition, T1 McDonald’s Omega (Ω) = 0.726, T2 McDonald’s Omega (Ω) = 0.278; T1 CR = 0.81, T1 AVE = 0.39; T2 CR = 0.81, T2 AVE = 0.41. Factor analysis showed that Comparative Fit Index (CFI) = 0.746, Tucker Lewis Index (TLI) = 0.724, Root Mean Square Error of Approximation (RMSEA) = 0.066.

*Emotion regulation*. We used the Self-Regulation Scale (SRS; Novak and Clayton, 2001) to assess participants’ emotion regulation. The SRS consists 26 items and three subscales (i.e., cognitive, emotional and behavioral regulation). The questionnaire is 4-point scale, from 1 (never) to 4 (always). In the study, we only used the emotional-regulation subscale which including 7 items (e.g., “*I have difficulty controlling my temper*”). Previous study in Chinese adolescents had proved the SRS was reliable and valid (Zhou et al., 2015). The internal reliabilities were 0.82 and 0.85 for the emotion-regulation at T1 and T2, respectively. In addition, T1 McDonald’s Omega (Ω) = 0.822, T2 McDonald’s Omega (Ω) = 0.848; T1 CR = 0.86, T1 AVE = 0.41; T2 CR = 0.88, T2 AVE = 0.45. Factor analysis showed that CFI = 0.855, TLI = 0.806, RMSEA = 0.057.

*Loneliness*. We used a self-report measure adapted from Asher et al. (1984) to assess participants’ loneliness. By using a 5-point (1 = not at all true, 5 = always true) scale, adolescents should respond to 16 statements (e.g., “*I’m always alone*”). The higher the score, the more lonely. Previous studies in Chinese adolescents had proved the measure was reliable and valid (Liu et al., 2021). The internal reliabilities were 0.93 and 0.92 for the loneliness at T1 and T2, respectively. In addition, T1 McDonald’s Omega (Ω) = 0.921, T2 McDonald’s Omega (Ω) = 0.919; T1 CR = 0.93, T1 AVE = 0.49; T2 CR = 0.93, T2 AVE = 0.47. Factor analysis showed that CFI = 0.896, TLI = 0.880, RMSEA = 0.091.

*Depression*. We used the Chinese version of the Children’s Depression Inventory (Kovacs, 1992) which consisting 14 items to assess participants’ depression. Each item provided three alternative responses (e.g., “*I’m not happy occasionally*,” “*I’m often unhappy*,” and “*I’m always unhappy*”), and participants should choose the best response according to their past 2 weeks life. The higher the score, the more depressed. Previous studies in Chinese adolescents had proved the measure was reliable and valid (e.g., Li et al., 2018). The internal reliabilities were 0.91 and 0.93 for the depression at T1 and T2, respectively. In addition, T1 McDonald’s Omega (Ω) = 0.913, T2 McDonald’s Omega (Ω) = 0.928; T1 CR = 0.88, T1 AVE = 0.35; T2 CR = 0.89, T2 AVE = 0.38. Factor analysis showed that CFI = 0.913, TLI = 0.897, RMSEA = 0.061.

### Design

Because cross-sectional design cannot reveal the causal relationship between variables, thus, by using questionnaire survey, a 1-year longitudinal design was used to explore the mediating role of emotion regulation between the two values and emotional adjustment (i.e., loneliness and depression) in Chinese adolescents.

### Procedure

The Ethics Committee of Shanghai Normal University reviewed and approved the design of the current study. During school hours, we measured all questionnaires on one school day, and the questionnaires administered in paper-and-pencil form. Before data collection, we trained postgraduate students as research assistants, and we also obtained approvals of schools and written informed consent of parents and students. The research assistants introduced the study aims and promised to keep the participants’ answers confidential. During the data collection, research assistants gave more explanations to participants, make sure that participants can understand the procedures. There was no evidence that participants had difficulties to understand the measure items and procedures. Data at Time 1 were collected in November of 2019, and data at Time 2 were collected in November of 2020.

### Data analysis

Data were analyzed by using SPSS 23.0. First, a 2 (Gender) × 2 (Region) × 2 (Time) multivariate analysis of variance (MANOVA) was conducted to compare boys and girls from two regions at Time 1 and 2 in terms of the two values, emotion regulation, loneliness and depression. The result of Test Kolmogorov–Smirnov showed that our data was normality (*p* > 0.05). In addition, the MANOVA analysis in our study met the key assumptions: independent variables, univariate normality (Test Kolmogorov–Smirnov) and homoscedasticity, the assumption of equality of variances (Test Levene), i.e., *ps* > 0.05. Second, the Person correlation was used to explore relations between the study variables. Third, the mediating model was used to test the mediating role of emotion regulation between the two values and loneliness and depression.

## Results

### Common method bias test

We examined the common method bias by using Harman’s single-factor test. We conducted the exploratory factor analysis (EFA) for all study variables according to the rotated principal component, and EFA extracted 11 factors at both T1 and T2 whose eigenvalues were larger than 1. The first factor accounted for 22.24% at T1 and 21.94% at T2 respectively, which less than 40% of the variance, and suggesting that the study had no serious common method bias.

### Preliminary statistics

All study variables were entered into a 2(Time: T1/T2) × 2(Gender: boy/girl) × 2(Region: Shanghai/Qingdao) multivariate analysis of variance (MANOVA). Descriptive statistics were shown in Table 1. Time (*F*(4, 767) = 14.49, Wilks’ λ = 0.93, *p* < 0.001, η_p_^2^ = 0.07) and Region (*F*(4, 767) = 10.14, Wilks’ λ = 0.95, *p* < 0.001, η_p_^2^ = 0.05) effect were significant, other effects were nonsignificant. Follow-up univariate analyses revealed that scores of self-transcendence values at T1 were higher than that of T2, but scores of self-enhancement values at T1 were lower than that of T2. And scores of depression of students in Shanghai were higher than that of students in Qingdao.

**Table 1:** Means, standard deviations, and correlations among study variables (*N* = 863).

	1	2	3	4	5	6	7	8	9	10
1. T1_ST	1									
2. T1_SE	−0.53^**^	1								
3. T1_LO	−0.09^**^	0.03	1							
4. T1_DE	−0.05	0.06	0.64^**^	1						
5. T1_ER	0.11^**^	−0.22^**^	−0.39^**^	−0.58^**^	1					
6. T2_ST	0.55^**^	−0.32^**^	−0.05	−0.01	0.07^*^	1				
7. T2_SE	−0.36^**^	0.58^**^	0.02	0.04	−0.17^**^	−0.48^**^	1			
8. T2_LO	−0.11^**^	0.06	0.66^**^	0.47^**^	−0.29^**^	−0.09^**^	0.06	1		
9. T2_DE	−0.06	0.07^*^	0.49^**^	0.68^**^	−0.41^**^	−0.01	0.10^**^	0.61^**^	1	
10. T2_ER	0.13^**^	−0.24^**^	−0.32^**^	−0.42^**^	0.60^**^	0.09^**^	−0.27^**^	−0.34^**^	−0.50^**^	1
*M*	0.18	−0.48	2.08	1.48	3.19	0.11	−0.33	2.10	1.47	3.16
SD	0.45	0.63	0.69	0.33	0.52	0.43	0.59	0.67	0.35	0.55

ST, self-transcendence; SE, self-enhancement; LO, loneliness; DE, depression; ER, emotion regulation. ^*^p < 0.05, ^**^p < 0.01.

As shown in Table 1, the Pearson correlation results mainly found that self-transcendence was negatively related to self-enhancement; loneliness and depression were negatively related to self-transcendence but positively related to self-enhancement; emotion regulation was positively correlated to self-transcendence, but negatively correlated to self-enhancement; emotion regulation was negatively related to loneliness and depression.

### The mediating effects analysis

Mplus7.0 was used to test the longitudinal mediation effect of emotion regulation at T2 between self-transcendence/self-enhancement values at T1 and loneliness and depression at T2, and gender, region and loneliness and depression at T1 were considered as controllable variables. In addition, according to Wen and Ye (2014), the indirect effect was significant but the total effect was not significant, indicating that the relationship between independent variable and dependent variable was suppressed by mediating variable.

### The relationship between self-transcendence and loneliness: Mediating role of emotion regulation

The regression results found that self-transcendence values at T1 negatively predicted loneliness at T2 (*β* = −0.08, *p* = 0.049). After adding emotion regulation at T2 into the regression equation, self-transcendence values at T1 did not negatively predict loneliness at T2 (*β* = −0.05, *p* = 0.204). In addition, self-transcendence values at T1 positively predicted emotion regulation at T2 (*β* = 0.17, *p* < 0.001), and emotion regulation at T2 negatively predicted loneliness at T2 (*β* = −0.18, *p* < 0.001), *R*^2^ = 0.406 (i.e., the proportion of variance explained).

**Figure 2:**
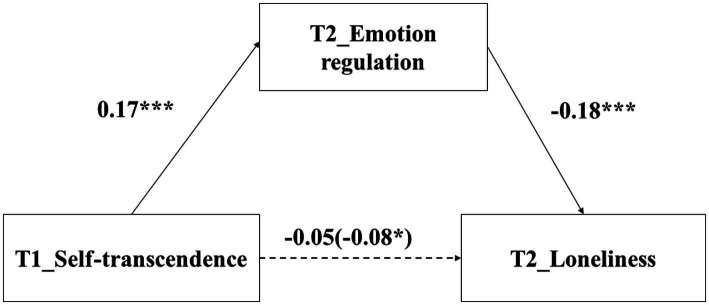
The mediating effect of emotion regulation. ^*^ *p* < 0.05, ^***^ *p* < 0.001.

The results of the mediating effect of emotion regulation at T2 were shown in Table 2 and Figure 2. Bootstrap test found that the indirect effect was 0.03 (R = 0.349, i.e., the ratio of indirect effect to total effect), *p* =. 004, 95% CI = [−0.05, −0.01], which suggested that emotion regulation at T2 mediated the relationship between self-transcendence values at T1 and loneliness at T2.

**Table 2:** Mediating effect analysis.

	Effect value	95%CI	
		LLCI	ULCI
Total effect	−0.08	−0.17	−0.003
Direct effect	−0.05	−0.14	0.03
Indirect effect	−0.03	−0.05	−0.01

### The relationship between self-transcendence and depression: Mediating role of emotion regulation

The regression results found that self-transcendence values at T1 did not predict depression at T2, *β* = −0.02, *p* = 0.358. After adding emotion regulation at T2 into the regression equation, self-transcendence values at T1 did not predict depression at T2 (*β* = 0.01, *p* = 0.521). In addition, self-transcendence values at T1 positively predicted emotion regulation at T2 (*β* = 0.16, *p* < 0.001), and emotion regulation at T2 negatively predicted depression at T2 (*β* = −0.18, *p* < 0.001).

**Figure 3:**
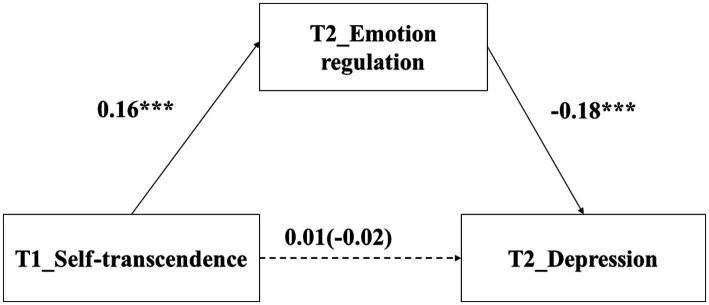
The mediating effect of emotion regulation. ^***^ *p* < 0.001.

The Bootstrap test found that the indirect effect was 0.03, *p* = 0.001, 95%CI = [−0.05, −0.01]. According to Wen and Ye (2014), these results indicated that the relationship between self-transcendence values and depression was suppressed by emotion regulation (see Figure 3).

### The relationship between self-enhancement and loneliness: Mediating role of emotion regulation

The regression results found that self-enhancement values at T1 did not predict loneliness at T2, *β* = 0.04, *p* = 0.15. After adding emotion regulation at T2 into the regression equation, self-enhancement values at T1 did not predict loneliness at T2 *β* = 0.005, *p* = 0.521. In addition, self-enhancement values at T1 negatively predicted emotion regulation at T2 (*β* = −0.21, *p* < 0.001), and emotion regulation at T2 negatively predicted loneliness at T2 (*β* = −0.18, *p* < 0.001).

**Figure 4:**
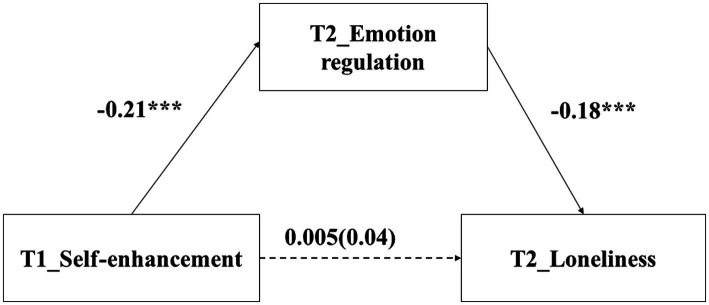
The mediating effect of emotion regulation. ^***^ *p* < 0.001.

The Bootstrap test found that the indirect effect was 0.04, *p* < 0.001, 95%CI = [0.02, 0.06]. According to Wen and Ye (2014), these results indicated that the relationship between self-enhancement values and loneliness was suppressed by emotion regulation (see Figure 4).

### The relationship between self-enhancement and depression: Mediating role of emotion regulation

The regression results found that self-enhancement values at T1 did not predict depression at T2, *β* = 0.02, *p* = 0.138. After adding emotion regulation at T2 into the regression equation, self-enhancement values at T1 did not predict depression at T2, *β* = −0.02, *p* = 0.254. In addition, self-enhancement values at T1 negatively predicted emotion regualtion at T2 (*β* = −0.21, *p* < 0.001), and emotion regulation at T2 negatively predicted depression at T2 (*β* = −0.18, *p* < 0.001).

**Figure 5:**
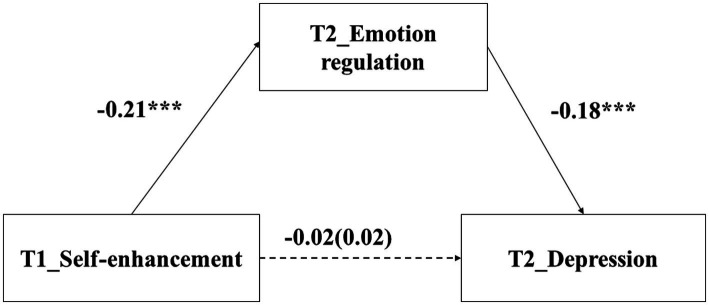
The mediating effect of emotion regulation. ^***^ *p* < 0.001.

The Bootstrap test found that the indirect effect was 0.04, *p* < 0.001, 95%CI = [0.03, 0.06]. According to Wen and Ye (2014), these results indicated that the relationship between self-enhancement values and depression was suppressed by emotion regulation (see Figure 5).

## Discussion

Previous studies have suggested that values have an important influence on emotional adjustment, little is known about its potential mediation mechanism. The findings of the present study showed that emotion regulation played a very important role in values and emotional adjustment among adolescents. The study contributes to our understanding of how values related to emotional adjustment, and also provides more evidences to understand relevant theories about values and emotion regulation.

### Temporal changes and region differences in adolescents’ values, emotional adjustment, and emotion regulation

The present study found that from T1 to T2, adolescents’ endorsement with self-transcendence values decreased and self-enhancement values increased. This finding is consistent with previous studies examining changes in adolescent values development (Schwartz, 2012; Benish-Weisman, 2019). On one hand, adolescence is the period of transition from childhood to adulthood, the transitions include a search for independence and the assertion of personal opinions (Braams et al., 2015; Gruenenfelder-Steiger et al., 2016). On the other hand, the changes of adolescent values may be not only influenced by the rapid development of social and economic (Huang et al., 2021), but also by popular pop culture, such as celebrity talent shows which convey the importance of materialism and personal achievement (Uhls and Greenfield, 2012). According to Schwartz’s theory of basic human values, changes in the importance of individual values occur in an organized and coherent manner (Schwartz, 2015). Specifically, if the importance of one value increases in importance while another value with the opposite motivation remains stable, adolescents may be disturbed by the inherent inconsistency between the two values. Thus, as the importance of one value increases, the importance of values driven by conflicting motives may decrease (Bardi et al., 2014).

In addition, the depression scores of Shanghai adolescents were higher than those of Shandong adolescents. This may be due to the fact that for adolescents in Shanghai, they may be more influenced by the rapid social development and new and different stimuli, and are prone to psychological conflicts under the heavy academic burden, thus they may experience more depression. In addition, Shanghai parents may experience more depression because they are under more economic, social, life and family pressures, so parental depression may affect adolescent depression (Lewis et al., 2017). Finally, crowded living environment may be another risk factor for adolescent depression (Ho et al., 2017).

We did not find any significant differences on the two values among two samples in the present study. Qingdao is more influenced by Confucian culture, which emphasizes harmonious relationships, and requires adolescents to comply with authority (Chen et al., 2012). Meanwhile, over the past three decades. China has dramatically morphed into a highly competitive and market-oriented society, with economic and social development (Liu et al., 2018). Whether in Shandong or Shanghai, adolescents are required to learn new social skills such as self-direction, independence, and self-confidence, in order to adapt to the new environment and pursue success (Liu et al., 2018). Thus, adolescents from Shanghai and Shandong in the present study showed similar values endorsement. However, previous studies suggested that contextual factors may moderate the relationships between adolescents’ values and adjustment (Heim et al., 2019). For example, compared to adolescents in rural areas, adolescents in urban areas value uniqueness show better peer relationships and academic performance (Chen et al., 2012). Thus, it is necessary to explore the role of culture on values and adjustment in samples with large background differences, such as eastern and western cities in China.

### The relationships between values and emotional adjustment: The mediating role of emotion regulation

We only found that there was a significant mediating effect of emotion regulation between self-transcendence values and loneliness; that is, self-transcendence values predicted adolescents’ loneliness through emotion regulation. Adolescents who endorsed on self-transcendence values not only feel more social-engaged emotions, such as empathy and compassion (Persson and Kajonius, 2016; de Leersnyder et al., 2017), but also engage in more activities that contribute to harmonious relationships, such as pro-social behaviors (Benish-Weisman et al., 2019). Thus, good interpersonal connections reduce their loneliness. In addition, individuals who identify with self-transcendence values tend to concern for the well-being of others and try to keep harmony with others and society (Dambrun and Ricard, 2011). In order to maintain friendly social relationships, they may be more proactive in regulating their emotions to prevent inappropriate emotional expressions which may cause negative effect on interpersonal relationships. Moreover, a lot of studies suggested that effective emotion regulation is related to mental health and well-being (Haines et al., 2016; Preece et al., 2021). In general, self-transcendence values contribute to regulate one’s emotion effectively, and effective emotion regulation further reduces one’s loneliness.

In addition, our results may suggest that emotion regulation may play a suppressed role between self-transcendence values and depression, between self-enhancement values and loneliness and depression. That is, although the relationships between values and emotional adjustment are not unrelated, their relationship may be linked through emotion regulation. Traditional tests for mediating effects assume that the independent and dependent variables are significantly correlated and that the total effect of the independent variable on the dependent variable is also significant. However, it has been suggested that the absence of a significant correlation between variables does not negate the causal relationship (Hayes, 2013). In other words, the prerequisite for the mediating effect test does not require a significant correlation between the variables (Rucker et al., 2011). In this study, the total and direct effect are not significant, but the indirect effect is significant. According to the new procedure for testing mediating effects (Wen and Ye, 2014), if the indirect effect is significant but the total effect is not, the final result should be interpreted as the suppressed effect.

Some possible reasons are explained as follows. First, the present study used two higher-order values, and it is possible that lower-order values may be more strongly associated with depression. One study found that power values were positively related to depression (Maercker et al., 2015), while benevolence values were negatively related to depression (Mousseau et al., 2014). Sagiv and Schwartz (2022) also noted that future examination of the relationship between values and variables requires consideration of which value categories (e.g., 10 lower-order values, 4 higher-order values) are more effective predictors. Thus, the relations between lower-order values and emotional adjustment should be explored in the future. Second, there may be other moderating variables between values and emotional adjustment (Heim, 2019). For example, some researchers have suggested that the association between values and subjective well-being is influenced by the socioeconomic context of the country (Sortheix and Lonnqvist, 2015). Specifically, in developed countries, identifying with social-oriented values (e.g., benevolence values) will increase one’s life satisfaction, but identifying with personal-oriented values (e.g., achievement values) may decrease one’s life satisfaction. However, in developing countries, the more one identified with benevolence values, the lower their life satisfaction; while the more they identified with achievement values, the higher their life satisfaction. Sortheix and Schwartz (2017) further found that compared to high cultural egalitarian contexts, in low cultural egalitarian contexts, growth-orientation and personal-orientation values (e.g., openness to change values) have stronger positive associations with subjective well-being, while self-protective orientation and social-orientation values (e.g., conservation values) have stronger negative associations with subjective well-being; and while achievement and power values have weaker negative associations with subjective well-being in low cultural egalitarian contexts. The above findings are helpful to understand our results, future studies could further examine the moderating role of variables such as socioeconomic status in the relationship between values and emotional adjustment.

Some meaningful implications of the study are as follows. The study found that values and emotional adjustment are linked by emotion regulation, which provides more evidences to understand GAM and SSHM theories. Moreover, the study sheds lights on the intervention of values and mental health. On one hand, families, schools, and society should take some effective measures to cultivate adolescent’s self-transcendence values; on the other hand, adolescents can learn how to regulate their emotions by using adaptive regulation strategies. Overall, suitable values and adaptive emotion regulation are two significant factors for promotion of emotional adjustment among adolescents.

## Limitations and future directions

First, participants in Shanghai were surveyed by convenient sampling, which may influence the accuracy of results. Participants in future studies should be all chosen at random. Second, although the study is a longitudinal design, the follow-up time was short and two measurements were not enough to reveal the long-term effects between variables. Future studies should extend the follow-up time, which helps to ensure the results of reliability and validity. Third, all the data of this study was collected by self-report, and there may be subjective bias in the process of subjects filling it out. Therefore, it is necessary to expand the data sources, such as parents, teachers and peers. Forth, as mentioned above, there may be some moderating variables between values and emotional adjustment. Future studies need to explore the relationship between values and emotional adjustment was moderated by possible variables (e.g., SES), which may provide more evidences and references to understand the effect of values on adjustment among adolescents.

## Data availability statement

The original contributions presented in the study are included in the article/supplementary material, further inquiries can be directed to the corresponding author.

## Ethics statement

The studies involving human participants were reviewed and approved by the Ethics Committee of Shanghai Normal University. Written informed consent to participate in this study was provided by the participants’ legal guardian/next of kin.

## Author contributions

PL analyzed the data and wrote the manuscript. DL designed the study and revised the manuscript. SL and DC revised the manuscript. BM and PY collected the data. All authors contributed to the article and approved the submitted version.

## Funding

This study was supported by Key Innovation Project of Shanghai Municipal Education Commission (2019-01-07-00-02-E00005).

## Conflict of interest

The authors declare that the research was conducted in the absence of any commercial or financial relationships that could be construed as a potential conflict of interest.

## Publisher’s note

All claims expressed in this article are solely those of the authors and do not necessarily represent those of their affiliated organizations, or those of the publisher, the editors and the reviewers. Any product that may be evaluated in this article, or claim that may be made by its manufacturer, is not guaranteed or endorsed by the publisher.

## References

[ref1] Al-Yagon M. (2011). Adolescents’ subtypes of attachment security with fathers and mothers and self-perceptions of socioemotional adjustment. Psychology 2, 291–299. doi: 10.4236/psych.2011.24046

[ref2] Anderson C. A. Bushman B. J. (2002). Human aggression. Annu. Rev. Psychol. 53, 27–51. doi: 10.1146/annurev.psych.53.100901.135231 11752478

[ref3] Asher S. R. Hymel S. Renshaw P. D. (1984). Loneliness in children. Child Dev. 55, 1456–1464. https://psycnet.apa.org/doi/10.2307/1130015

[ref4] Bardi A. Buchanan K. E. Goodwin R. Slabu L. (2014). Value stability and change during self-chosen life transitions: self-selection versus socialization effects. J. Pers. Soc. Psychol. 106, 131–147. doi: 10.1037/a0034818, PMID: 24219783

[ref5] Benish-Weisman M. (2019). What can we learn about aggression from what adolescents consider important in life? The contribution of values theory to aggression research. Child Dev. Perspect. 13, 260–266. doi: 10.1111/cdep.12344

[ref6] Benish-Weisman M. Daniel E. Sneddon J. Lee J. (2019). The relations between values and prosocial behavior among children: the moderating role of age. Personal. Individ. Differ. 141, 241–247. doi: 10.1016/j.paid.2019.01.019

[ref7] Bojanowska A. Piotrowski K. (2018). Values and psychological well-being among adolescents – are some values ‘healthier’ than others? Eur. J. Dev. Psychol. 16, 402–416. doi: 10.1080/17405629.2018.1438257

[ref06] Braams B. R. Van Duijvenvoorde A. C. Peper J. S. Crone E. A. (2015). Longitudinal changes in adolescent risk‐taking: A comprehensive study of neural responses to rewards, pubertal development, and risk‐taking behavior. Journal of Neuroscience 35, 7226–7238. doi: 10.1523/JNEUROSCI.4764-14.2015 25948271 PMC6605271

[ref9] Casey B. J. Jones R. M. Levita L. Libby V. Pattwell S. S. Ruberry E. J.. (2010). The storm and stress of adolescence: insights from human imaging and mouse genetics. Dev. Psychobiol. 52, 225–235. doi: 10.1002/dev.20447, PMID: 20222060 PMC2850961

[ref11] Chen X. Wang L. Liu J. (2012). “Adolescent cultural values and adjustment in the changing Chinese society” in Values, Religion, and Culture in Adolescent Development. eds. Trommsdorff G. Chen X. (Cambridge: Cambridge University Press), 235–252.

[ref12] Cieciuch J. Schwartz S. H. (2012). The number of distinct basic values and their structure assessed by PVQ-40. J. Pers. Assess. 94, 321–328. doi: 10.1080/00223891.2012.655817, PMID: 22329443

[ref13] Cieciuch J. Schwartz S. H. Davidov E. (2015). “Values, social psychology of,” in International Encyclopedia of the Social and Behavioral Sciences. ed. Wright J. D.. 2nd ed (Oxford: Elsevier), 41–46.

[ref15] Dambrun M. Ricard M. (2011). Self-centeredness and selflessness: a theory of self-based psychological functioning and its consequences for happiness. Rev. Gen. Psychol. 15, 138–157. doi: 10.1037/a0023059

[ref02] Dambrun M. (2017). Self-centeredness and selflessness: happiness correlates and mediating psychological processes. PeerJ 5:e3306. doi: 10.7717/peerj.3306 28507820 PMC5429736

[ref17] de Leersnyder J. Koval P. Kuppens P. Mesquita B. (2017). Emotions and concerns: situational evidence for their systematic co-occurrence. Emotion 18, 597–614. doi: 10.1037/emo0000314, PMID: 28604034

[ref18] Gruenenfelder-Steiger A. E. Harris M. A. Fend H. A. (2016). Subjective and objective peer approval evaluations and self-esteem development: a test of reciprocal, prospective, and long-term effects. Dev. Psychol. 52, 1563–1577. doi: 10.1037/dev0000147, PMID: 27690495

[ref19] Gu X. Tse C.-H. (2018). Abstractness and desirableness in the human values system: self-transcendence values are construed more abstractly, but felt more closely than are self-enhancement values. Asian J. Soc. Psychol. 21, 282–294. doi: 10.1111/ajsp.12335

[ref20] Haines S. J. Gleeson J. Kuppens P. Hollenstein T. Ciarrochi J. Labuschagne I.. (2016). The wisdom to know the difference: strategy-situation fit in emotion regulation in daily life is associated with well-being. Psychol. Sci. 27, 1651–1659. doi: 10.1177/0956797616669086, PMID: 27738099

[ref21] Hayes A. F. (2013). Introduction to Mediation, Moderation, and Conditional Process Analysis: A Regression-Based Approach. New York: Guilford Press.

[ref22] Heim E. (2019). Value orientations and mental health: a theoretical review. Transcult. Psychiatry 56, 449–470. doi: 10.1177/1363461519832472, PMID: 30924415

[ref23] Heim E. Maercker A. Boer D. (2019). Value orientations and mental health: a theoretical review. Transcult. Psychiatry 56, 449–470. doi: 10.1177/1363461519832472, PMID: 30924415

[ref24] Ho H. C. Lau K. Ka L. Yu R. Wang D. Woo J.. (2017). Spatial variability of geriatric depression risk in a high-density city: a data-driven socio-environmental vulnerability mapping approach. Int. J. Environ. Res. Public Health 14:994. doi: 10.3390/ijerph14090994, PMID: 28858265 PMC5615531

[ref25] Huang Z. Jing Y. Yu F. Gu Y. Zhou X. Zhang J.. (2021). Increasing individualism and decreasing collectivism? Cultural and psychological change around the globe. Adv. Psychol. Sci. 26, 2068–2080. doi: 10.3724/SP.J.1042.2018.02068

[ref26] Inglehart R. (1978). The Silent Revolution: Changing Values and Political Styles Among Western Publics. Princeton, NJ: Princeton University Press.

[ref28] Kovacs M. (1992). The Children’s Depression Inventory (CDI) Manual. Toronto, ON: MultiHealth Systems.

[ref04] Lewis G. Neary M. Polek E. Flouri E. Chen M. X. Lewis G. (2017). The association between paternal and adolescent depressive symptoms: evidence from two population-based cohorts. Lancet Psychiatr 4, 920–926. doi: 10.1016/S2215-0366(17)30408-X 29153626

[ref29] Li D. Zhou T. Liu J. S. Dai Y. Chen M. X. Chen X. Y. (2018). Values of adolescent across regions in China: relations with social, school, and psychological adjustment. J. Psychol. Sci. 41, 1292–1301. doi: 10.16719/j.cnki.1671-6981.20180603

[ref30] Liu X. Fu R. Li D. Liu J. Chen X. (2018). Self- and group-orientations and adjustment in urban and rural Chinese children. J. Cross-Cult. Psychol. 49, 1440–1456. doi: 10.1177/0022022118795294

[ref31] Liu P. Wang X. Li D. Zhang R. Li H. Han J. (2021). The benefits of self-transcendence: examining the role of values on mental health among adolescents across regions in China. Front. Psychol. 12:630420. doi: 10.3389/fpsyg.2021.630420, PMID: 33679555 PMC7925830

[ref32] Liu P. Zhang R. Li D. (2022). The effect and mechanisms of self-transcendence values on durable happiness. Adv. Psychol. Sci. 30, 660–669. doi: 10.3724/SP.J.1042.2022.00660

[ref33] Maercker A. Zhang X. C. Gao Z. Kochetkov Y. Lu S. Sang Z.. (2015). Personal value orientations as mediated predictors of mental health: a three-culture study of Chinese, Russian, and German university students. Int. J. Clin. Health Psychol. 15, 8–17. doi: 10.1016/j.ijchp.2014.06.001, PMID: 30487817 PMC6224790

[ref05] Mousseau A. C. Scott W. D. Estes D. (2014). Values and depressive symptoms in American Indian youth of the northern plains: Examining the potential moderating roles of outcome expectancies and perceived community values. Journal of Youth and Adolescent 43, 426–436. doi: 10.1007/s10964-013-9982-9 23857243

[ref34] Novak S. P. Clayton R. R. (2001). The influence of school environment and self-regulation on transitions between stages of cigarette smoking: a multi-level analysis. Health Psychol. 20, 196–207. doi: 10.1037/0278-6133.20.3.196, PMID: 11403217

[ref35] Persson B. N. Kajonius P. J. (2016). Empathy and universal values explicated by the empathyaltruism hypothesis. J. Soc. Psychol. 156, 610–619. doi: 10.1080/00224545.2016.1152212, PMID: 26885864

[ref36] Preece D. A. Goldenberg A. Becerra R. Boyes M. Gross J. J. (2021). Loneliness and emotion regulation. Personal. Individ. Differ. 180:110974. doi: 10.1016/j.paid.2021.110974

[ref37] Rokeach M. (1973). The Nature of Human Values. New York, NY: Free Press.

[ref38] Rottenberg J. Gross J. J. (2007). Emotion and emotion regulation: a map for psychotherapy researchers. Clin. Psychol. Sci. Pract. 14, 323–328. doi: 10.1111/j.1468-2850.2007.00093.x

[ref39] Rucker D. D. Preacher K. J. Tormala Z. L. Petty R. E. (2011). Mediation analysis in social psychology: current practice and new recommendations. Personal. Soc. Psychol. Compass 5, 359–371. doi: 10.1111/j.1751-9004.2011.00355.x

[ref40] Sagiv L. Schwartz S. H. (2022). Personal values across cultures. Annu. Rev. Psychol. 73, 517–546. doi: 10.1146/annurev-psych-020821-125100 34665670

[ref41] Schwartz S. H. (1992). Universals in the content and structure of values: theoretical advances and empirical tests in 20 countries. Adv. Exp. Soc. Psychol. 25, 1–65. doi: 10.1016/S0065-2601(08)60281-6

[ref03] Schwartz S. H. Bardi S. (2001). Value hierarchies across cultures: taking a similarities perspective. J. Cross Cult. Psychol. 73, 517–546. doi: 10.1146/annurev-psych-020821-125100

[ref42] Schwartz S. H. (2012). “Values and religion in adolescent development: cross-national and comparative evidence,” in Values, Religion, and Culture in Adolescent Development. eds. Trommsdorff G. Chen X. (Cambridge, England: Cambridge University Press), 97–122.

[ref43] Schwartz S. H. (2014). Functional theories of human values: comment on Gouveia, Milfont, and Guerra (2014). Personal. Individ. Differ. 68, 247–249. doi: 10.1016/j.paid.2014.03.024

[ref44] Schwartz S. H. (2015). “Basic individual values: sources and consequences,” in Handbook of Value. eds. Sander D. Brosch T. (Oxford: UK, Oxford University Press).

[ref45] Schwartz S. H. Rubel T. (2005). Sex differences in value priorities: Crosscultural and multimethod studies. J. Pers. Soc. Psychol. 89, 1010–1028. doi: 10.1037/0022-3514.89.6.1010, PMID: 16393031

[ref46] Song J. H. Colasante T. Malti T. (2018). Helping yourself helps others: linking children’ emotion regulation to prosocial behavior through sympathy and trust. Emotion 18, 518–527. doi: 10.1037/emo0000332, PMID: 28581324

[ref47] Sortheix F. M. Lonnqvist J. E. (2015). Personal value priorities and life satisfaction in Europe: the moderating role of socioeconomic development. J. Cross-Cult. Psychol. 45, 282–299. doi: 10.1177/0022022113504621

[ref48] Sortheix F. M. Schwartz S. H. (2017). Values that underlie and undermine well-being: variability across countries. Eur. J. Personal. 31, 187–201. doi: 10.1002/per.2096

[ref01] Strayer J. Roberts W. (2010). Empathy and observed anger and aggression in five-year-olds. Social Development 13, 1–13. doi: 10.1111/j.1467-9507.2004.00254.x

[ref49] Tamir M. Schwartz S. H. Cieciuch J. Riediger M. Torres C. Scollon C.. (2016). Desired emotionsacross cultures: a value-based account. J. Pers. Soc. Psychol. 111, 67–82. doi: 10.1037/pspp0000072, PMID: 26524003

[ref50] Twenge J. M. (2015). Time period and birth cohort differences in depressive symptoms in the US, 1982–2013. Soc. Indic. Res. 121, 437–454. doi: 10.1007/s11205-014-0647-1

[ref51] Uhls Y. T. Greenfield P. M. (2012). The value of fame: preadolescent perceptions of popular variability across countries. Eur. J. Personal. 48, 315–326. doi: 10.1037/a0026369 22182297

[ref52] Wen Z. Ye B. (2014). Analyses of mediating effects: the development of methods and models. Adv. Psychol. Sci. 22, 731–745. doi: 10.3724/SP.J.1042.2014.00731

[ref53] Zhou Y. Bullock A. Liu J. Cheah C. (2015). Validation of the self-regulation scale in Chinese children. J. Psychoeduc. Assess. 34, 589–594. doi: 10.1177/0734282915622853

